# The Impact of Potassium Channel Gene Polymorphisms on Antiepileptic Drug Responsiveness in Arab Patients with Epilepsy

**DOI:** 10.3390/jpm8040037

**Published:** 2018-11-14

**Authors:** Laith N. AL-Eitan, Islam M. Al-Dalalah, Afrah K. Elshammari, Wael H. Khreisat, Ayah Y. Almasri

**Affiliations:** 1 Department of Applied Biological Sciences, Jordan University of Science and Technology, Irbid 22110, Jordan; imaldalalah15@sci.just.edu.jo (I.M.A.-D.); aya_almasri@outlook.com (A.Y.A.); 2 Department of Biotechnology and Genetic Engineering, Jordan University of Science and Technology, Irbid 22110, Jordan; 3 Queen Rania hospital for children, King Hussein Medical Centre, Royal medical services (RMS), Amman 11118, Jordan; khlifafrah@yahoo.com (A.K.E.); wael_khreisat@yahoo.com (W.H.K.)

**Keywords:** epilepsy, potassium channels, pharmacogenetics, antiepileptic drug

## Abstract

This study aims to investigate the effects of the three potassium channel genes *KCNA1*, *KCNA2*, and *KCNV2* on increased susceptibility to epilepsy as well as on responsiveness to antiepileptic drugs (AEDs). The pharmacogenetic and case-control cohort (*n* = 595) consisted of 296 epileptic patients and 299 healthy individuals. Epileptic patients were recruited from the Pediatric Neurology clinic at the Queen Rania Al Abdullah Hospital (QRAH) in Amman, Jordan. A custom platform array search for genetic association in Jordanian-Arab epileptic patients was undertaken. The MassARRAY system (iPLEX GOLD) was used to genotype seven single nucleotide polymorphisms (SNPs) within three candidate genes (*KCNA1*, *KCNA2*, and *KCNV2*). Only one SNP in *KCNA2*, rs3887820, showed significant association with increased risk of susceptibility to generalized myoclonic seizure (*p*-value < 0.001). Notably, the rs112561866 polymorphism of the *KCNA1* gene was non-polymorphic, but no significant association was found between the *KCNA1* (rs2227910, rs112561866, and rs7974459) and *KCNV2* (rs7029012, rs10967705, and rs10967728) polymorphisms and disease susceptibility or drug responsiveness among Jordanian patients. This study suggests that a significant association exists between the *KCNA2* SNP rs3887820 and increased susceptibility to generalized myoclonic seizure. However, the present findings indicate that the *KCNA1* and *KCNV2* SNPs do not influence disease susceptibility and drug responsiveness in epileptic patients. Pharmacogenetic and case-control studies involving a multicenter and multiethnic approach are needed to confirm our results. To improve the efficacy and safety of epilepsy treatment, further studies are required to identify other genetic factors that contribute to susceptibility and treatment outcome.

## 1. Introduction

Epilepsy is a neurological disorder that affects people of all ages and is a considerable cause of morbidity and mortality [1]. Characterized by abnormal central nervous system electrical activity and recurrent seizures, epilepsy is one of the most commonly reported neurological conditions [2]. Epileptic seizures can be classified into generalized epileptic seizures (GE), which are characterized by discharges arising simultaneously from both cerebral hemispheres, and partial epileptic seizures (PE), in which the discharges arise from focal cortical disturbances. Generalized epileptic seizures can be further sub-divided into two types: generalized myoclonic seizures (GM) and generalized tonic-clonic seizures (GTC) [3,4].

Currently, epilepsy has been associated with at least 33 chromosomal regions, and experimental studies have shown that mutations in these regions lead to neurological dysfunction, especially if they encode for ion channel proteins [5,6]. Ion channels are associated with many cellular functions and are directly or indirectly changed in the majority of pathological conditions, leading to channelopathies [7]. Voltage-gated potassium ion channels (Kv) are extensively distributed in both the central and peripheral nervous systems where they participate in various processes [8]. Mutations that result in Kv gene dysfunction have been linked with epilepsy and other disorders that exhibit seizures [6]. *KCNA1* and *KCNA2* are essential Kv genes which encode the mammalian Kv1.1 and Kv1.2 channels, respectively, while the *KCNV2* gene encodes Kv8.2, a regional overlay consisting of Kv2 subunits as functional heterotetramers [9]. Dysfunctional mutations of the aforementioned Kv genes have been identified in the context of generalized epileptic pathology [10,11].

Multiple heterozygous *KCNA1* point mutations have been associated with generalized or partial seizures in episodic ataxia type 1 [6,12]. Moreover, de novo loss- or gain-of-function mutations in the *KCNA2* gene lead to myoclonic epilepsy, epileptic encephalopathy, and ataxia [13,14]. A study conducted on the Kv1.2 knockout mouse model observed that *KCNA2* loss-of-function mutations result in a greater susceptibility to seizures [15]. In contrast, *KCNV2* mutations are related to febrile and afebrile partial seizures and cause the voltage dependence of the channel to fail [15,16].

Although numerous studies have been conducted on the pharmacogenetics (PGx) of epilepsy, there is no conclusive data to explain drug response capacity and to inform treatment decisions [17,18,19]. Antiepileptic drugs (AEDs) reduce epileptic seizures by targeting Kv, but it has been found that variants of drug transporter (*ABCB1*, *ABCC2*) and ion channel (*SCN1A*, *SCN2A*) genes influenced AED efficacy and contributed to drug-resistant epilepsy [20,21,22].

To the best of our knowledge, no pharmacogenetic study has evaluated the role of the *KCNA1*, *KCNA2*, and *KCNV2* genes in epilepsy susceptibility in the Jordanian-Arab population. With these concepts in mind, we try to understand the role of AED pharmacology in the context of inter individual genetic variation. We direct our attention to the possible influence of the *KCNA1*, *KCNA2*, and *KCNV2* genes on the pathogenesis of epilepsy and responsiveness to treatment involving common drugs.

## 2. Material and Methods

### 2.1. Study Population and Design

The pharmacogenetic and case-control cohort consisted of 595 participants (296 epileptic patients and 299 healthy individuals) recruited between the period of 2017 to 2018 from the Pediatric Neurology Clinic at Queen Rania Al Abdullah Hospital (QRAH) and the Blood Bank at the Jordanian Royal Medical Services (JRMS). Using the guidelines of the International League Against Epilepsy and other standardized protocols, patients were classified as either generalized (GE) or partial epileptics (PE) [3,23]. The former seizure type, GE, was further classified as generalized myoclonic epileptic (GME) or generalized tonic-clonic (GTC) epileptic seizures. Inclusion criteria for this study involved being under the age of 15 years old, having had at least two attacks of seizure in under 24 h in the last six months, and receiving AED treatment for at least three months. Participants were also required to have normal psychometric development, normal neurologic examination, and normal background activity. In contrast, patients were excluded from this study if they lacked medical records, did not have a reliable seizure frequency, were not compliant with AED treatment, suffered from liver disorders, did not provide written consent or did not visit the clinic regularly.

**Figure 1:**
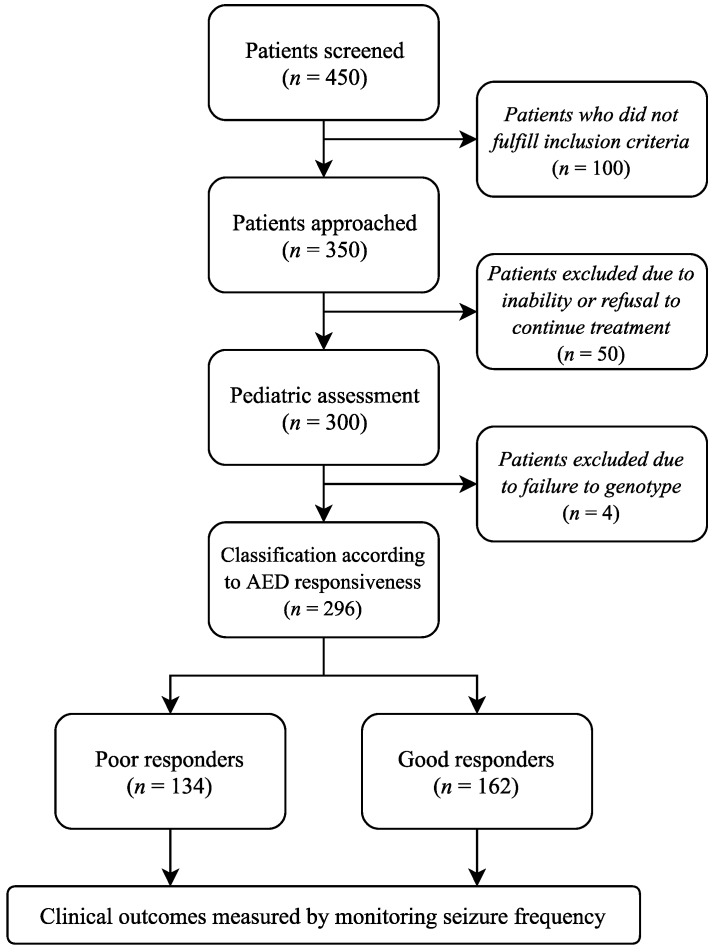
Flow chart of epileptic patients (*N* = 296).

Initially, 450 patients were screened, but only 350 fulfilled the inclusion criteria and participated in this study (Figure 1). Of the 350 patients, 50 patients could not complete the treatment program for clinical reasons or refusal to continue, meaning that 300 patients agreed to be part of this study. A further four patients were then excluded from the final analysis due to a failure in genotyping. In total, complete data regarding AED intake were obtained from 296 patients.

### 2.2. Study Design

A semi-standardized baseline survey was designed to collect demographic and clinical data from patient medical records. Informed consent was obtained from all subjects, and the study protocol was approved by both the Human Ethics Committee at Jordan University of Science and Technology and the Jordanian Royal Medical Services Hospital in Amman, Jordan (ethical approval code number 16/111/2017). These data include information about age, gender, body mass index, and other lifestyle characteristics. In addition, patient medical records were used to collect clinical and phenotypic data about the epilepsy patients. These included data on clinical factors that have been previously associated with epilepsy therapy, including primary indication for AED therapy, electroencephalograms, magnetic resonance imaging scans, mean daily AED doses, use of concomitant medication, kidney function tests, liver function test, lipid profiles, cell blood count (CBC), vitamin D concentration, and thyroid function test. Information on adverse events such as rash, allergy, or weight fluctuation was also collected.

### 2.3. Treatment Approach

All subjects in this study received AED therapy for at least two years. Following the standard practice at the Pediatric Neurology Clinic at the QRAH, the antiepileptic protocol began with 10 mg/kg of Convulex (G.L. Pharma GmbH, Lannach, Austria) for patients diagnosed with GE or 5 mg/kg daily of Tegretol (Novartis Pharmaceuticals UK Ltd., Surrey, England) for patients diagnosed with PE. Seizure frequency monitoring for the first three to four weeks after initiation of therapy to ensure that the dose was effective. During follow-up visits, patients were given the therapeutic dose by increasing the initial Convulex and Tegretol doses to 20 mg/kg and 10 mg/kg, respectively, in order to minimize seizure frequency. Healthy Jordanian-Arab controls were recruited from the Jordanian Royal Medical Services in Jordan.

### 2.4. Outcome Measure

The frequency of seizure monitoring for dose adjustments was determined by the physician based on the recommendations of the International League Against Epilepsy as well as family observations [3,23]. Patients were classified based on their responsiveness to AEDs, dividing them into good and poor responders (Figure 1). Good responders are those patients who require the lowest AED doses or who have taken only one drug without relapse in the past six months. In contrast, poor responders are those patients who require the highest AED doses or who have taken more than one drug.

### 2.5. SNP Selection and Genotyping

After blood samples were collected, DNA was extracted using the Wizard Genomic DNA Purification Kit (Promega Corporation, Madison, WI, USA). Seven SNPs within the *KCNA1*, *KCNA2,* and *KCNV2* genes were selected from public databases such as the SNP database of the National Center for Biotechnology Information (NCBI, Bethesda, MD, USA) (http://www.ncbi.nlm.nih.gov/SNP/), Ensembl database (http://www.ensembl.org/index.html) and the Applied Biosystems SNP database (http://www.appliedbiosystems.com). The list of genes, their SNP IDs, and their location on chromosomes are shown in Table S1. The MassARRAY (iPLEX GOLD) system (Sequenom, San Diego, CA, USA) was utilized by the Australian Genome Research Facility (AGRF) for genotyping as stated by the manufacturer’s recommendations.

### 2.6. Statistical Analysis

Several statistical genetic association analyses, including haplotype analysis, were conducted to test which of the selected SNPs were related to epilepsy susceptibility and response to AEDs. The SNPStats Web Tool was used to perform all analyses, including the Hardy-Weinberg equilibrium (HWE) values for the genotype distribution and the minor allelic frequency (MAF) (https://www.snpstats.net/start.htm).

## 3. Results

### 3.1. Sample Characteristics

A total of 595 subjects were included in our study, which consisted of 299 healthy controls and 296 Jordanian (Arab) epilepsy patients. One hundred and sixty-two patients (54.7%) with good responder status and one hundred and thirty-four patients (45.3%) with poor responsiveness were identified based on the guidelines of the Pediatric Neurology Clinic at QRAH. Patients’ demographic and clinical data as well as the demographic data of healthy controls are summarized in Tables S2–S4, respectively. There were no differences between patients with good response and patients with poor response in terms of age, gender, age of onset, and in subgroup distribution. All the aforementioned data are shown in Table S2.

### 3.2. Hardy-Weinberg Equilibrium Test

All SNP distributions in epileptics and healthy controls, which were in HWE, are summarized in Table S5. Notably, one of the *KCNA1* SNPs (rs112561866) was non-polymorphic, while the remaining two *KCNA1* SNPs (rs2227910 and rs7974459), one *KCNA2* SNP (rs3887820), and three *KCNV2* SNPs (rs7029012, rs10967705 and rs10967728) were included in the study.

### 3.3. Quality Control

All genotyped SNPs were tested for HWE and Mendelian errors. All duplicates were identical, the water controls were clean, the markers were consistent with HWE and no Mendelian errors were found. In total, seven SNPs (100%) passed the quality control for performance genotyping. All seven SNPs were analyzed with the MassARRAY sequencing system (iPLEX GOLD), and the genotypes were achieved with great accuracy and an average success rate of 98%. The mean genotypic discrepancy rate (±SD) across the seven loci was only 0.03% (±0.02%) in the entire cohort (595 subjects). An example of the results of the Sequenom iPLEX Gold test is shown in Figure S1, depicting a representative scatter plot for the *KCNV2* rs10967728 SNP that is stained for different genotype calls: GG (green), GC (yellow) and CC (blue) and no call (red).

### 3.4. Allelic and Genotypic Distribution in Epileptic Patients

Comparisons between epilepsy patients and healthy controls revealed no differences in the distributions of all investigated SNPs. Table 1 exhibits the genotypic and allelic frequencies for the total 296 patient and 299 control samples. Moreover, this study found that there are no genetic differences in allelic and genotypic distributions based on gender variations (data not shown) in all studied SNPs except one SNP (rs2227910) within *KCNA1* gene with a *p* < 0.023.

### 3.5. Association of KCNA1, KCNA2, and KCNV2 SNPs with Susceptibility to Generalized Epilepsy

In addition, we further classified epileptic patients into those with generalized (172) and partial (124) epilepsy. Then, we analyzed the SNP distributions of generalized epilepsy (GE) patients, which showed that the rs3887820 SNP of the *KCNA2* gene was associated with susceptibility to GE. The frequency of the *KCNA2* rs3887820 CA + AA genotypes were higher in GE patients than that in healthy controls (49.6% vs. 17.1%, odds ratio (OR) = 4.75 (3.09 − 7.28), *p* = < 0.0001) (Table 2). Moreover, we divided patients with GE into those with GM (*n* = 115) and GTC (*n* = 57), after which we tested the SNP distributions of the subgroups. The results showed that there are no significant differences between the GE subgroups and the healthy controls (Table 3). Moreover, there were no significant *KCNA1* and *KCNV2* haplotypes associated with GE risk (Table S6).

### 3.6. Association of SNPs KCNA1, KCNA2 and KCNV2 Genotypes with Partial Epilepsy Susceptibility

In case of the PE patients, there were no significant differences between the SNP distributions of 124 PE patients and 299 healthy controls (Table 4).

### 3.7. Association of SNPs KCNA1, KCNA2, and KCNV2 Genotypes with Epilepsy Patients Responsiveness

We analyzed the distributions of *KCNA1*, *KCNA2,* and *KCNV2* loci in 134 patients with poor responder status and 162 patients with good responder status. The frequency of the *KCNV2* rs10967728 CC genotype was higher in patients with poor response than in those with good response (23.7% vs. 18.2%, OR = 1.39 (0.79 − 2.46), *p* = 0. 26) (Table 5). After analyzing the frequencies of Kv-related gene haplotypes in the poor and good responders, we found that a *KCNA1* and *KCNV2* haplotype did not show a significant association with epilepsy responsiveness (Table S7).

## 4. Discussion

Ion channels are associated with various cellular functions and are often changed when the body enters a pathological state [7]. Remarkably, potassium channels play a significant role in neuronal excitability and are generally correlated to the inward negative membrane potential [10]. However, the effects of the *KCNA1*, *KCNA2*, and *KCNV2* genes on epilepsy development and the effectiveness of AEDs are not yet clear. In this study, blood samples were collected from 299 healthy controls and 296 Jordanian Arab patients with epilepsy (consisting of 162 patients with good response and 134 patients with poor response), and seven *KCNA1*, *KCNA2*, and *KCNV2* SNPs. This study reported that the rs3887820 SNP of the *KCNA2* gene was associated with susceptibility to GME. In contrast, the remaining evaluated SNPs of the *KCNA1*, *KCNA2*, and *KCNV2* genes did not show any statistically significant relationship with the risk of epilepsy, its subtypes (partial, myoclonic and tonic colonic seizures), or with the patient’s response capacity.

For the association between potassium ion channels variants and increased risk of epilepsy and its subtypes, Jorge et al. (2011) identified unique nonsynonymous *KCNV* variants R7K and M285R in two unrelated epileptic children, leading to differences in clinical variability and a potential of improving Kv8.2-mediated suppression of Kv2.1 currents [16]. The Kv8.2 channel is encoded by the *KCNV2* gene, which is electro-physiologically silent when incorporated into the homotetramer and modifies the properties of the Kv2 and Kv3 channels to influence their properties and membrane translocation [10,16]. Kv8.2 with Kv2.1 contributes to the limited K+ current rectifier in the pyramidal neurons of the hippocampus, which is of particular importance for the onset of seizures [10]. In fact, patients with the R7K variant exhibited relatively febrile and afebrile seizures, while those with the M285R variant exhibited very poor responsiveness in the context of epileptic encephalopathy [16].

In addition to R7K and M285R, the *KCNV2* rs7029012 variant has also been associated with the risk of juvenile myoclonic epilepsy, as it can affect the binding of the acting element by inducing changes in *KCNV2* expression [9,16]. Our study found no association between the rs7029012 variant and susceptibility to epilepsy or epileptic responsiveness to AED treatment. Likewise, Qu et al. (2017) found that the *KCNV2* rs10967705 synonymous variant is associated with the risk of both juvenile myoclonic epilepsy and childhood absence epilepsy, hypothesizing that it was in linkage disequilibrium with rs7029012 [9]. However, the present study found no significant differences between rs10967705 and GMs, GTCs and PEs nor any significant linkage disequilibrium between rs10967705 and rs7029012. No association was observed between the rs7029012, rs10967705, and rs10967728 variants of the *KCNV2* gene and the risk of susceptibility to the various subtypes of epilepsy (Table 3, Table 4 and Table S6). Moreover, it has been reported that synonymous variants do not produce changes in gene expression level of the *KCNV2* gene. Therefore, they are not expected to change the function of the protein [24,25].

The *KCNA1* and *KCNA2* genes, which encode the Kv1 subfamilies and include the Kv1.1 and Kv1.2 subunits, respectively, play a significant role in action initiation and configuration in synapses, soma, axons, and proximal dendrites [10]. Diverse heterozygous point mutations of *KCNA1* with generalized or partial seizures have been reported in episodic type 1 ataxia, a neurological disorder characterized by attacks of generalized ataxia and spontaneous muscle tremors [6,12,26]. In contrast, rs112561866 was found to be non-polymorphic in the Jordanian population, and no association was observed between the rs2227910 and rs7974459 variants of the *KCNA1* gene. Contrasting with the previous study that reported that the *KCNA2* rs3887820 variant did not increase GE risk [9], the current study found a significant association between the *KCNA2* rs3887820 variant and susceptibility to GE of the myoclonic subtype (*p*-value < 0.0001) (Table 2 and Table 3).

Since resistance to AEDs is a major challenge for health care professionals, it is important to understand the association between potassium ion channel variants and AED responsiveness. Although multiple medications were used for treatment, some patients showed poor response to AED treatment. AEDs are directed to multiple but non-specific ion channels to perform pharmacological action, which develops complex electrophysiological state in brain neurons [9]. In our study, a pharmacogenetic analysis was also conducted to evaluate the association between the *KCNA1*, *KCNA2*, and *KCNV2* variants and resistance to epileptic drugs. We found that there are no significant differences between all investigated variants in the *KCNA1*, *KCNA2*, and *KCNV2* genes and drug responsiveness. Moreover, there was no significant association between the *KCNA1* SNP haplotype and drug resistance nor with the *KCNV2* SNP haplotype (Table 5 and Table S7).

This study is the first pharmacogenetic association study of *KCNA1*, *KCNA2*, and *KCNV2* polymorphisms in Jordanian epileptics. It was found that the rs3887820 SNP in the *KCNA2* gene was associated with generalized myoclonic seizure. On the other hand, this study found that there is no significant effect of the rs2227910, rs112561866, and rs7974459 SNPs within the *KCNA1* gene and the rs7029012, rs10967705, and rs10967728 SNPs within the *KCNV2* gene on susceptibility to epilepsy and drug responsiveness among Jordanian patients. Further studies are needed to identify the other genetic factors that contribute to genetic susceptibility and treatment outcomes to improve the efficacy and safety of epilepsy treatment. Investigating genes in other ion channels (sodium and calcium) should also be conducted. The multivariate associations analysis method (which concurrently investigate the contribution of multiple genes) is necessary for the understanding the effect of the genetic makeup on response to AEDs. Future studies may also be directed towards using haplotype analysis and include a cohort with more specific clinical phenotypic data.

## Figures and Tables

**Table 1:** The distributions of Kv-associated single nucleotide polymorphisms (SNPs) in 296 epileptic patients and 299 healthy controls.

Gene	SNP ID	Model	Cases (%)	Controls (%)	OR (95% CI)	*p*-Value *
***KCNA1***	**rs2227910**	CC/GC/GG	41.1/40.4/18.5	38.6/45.0/16.4	_	0.52
		CC/(GC + GG)	41.1/58.9	38.6/61.4	0.90 (0.65−1.25)	0.53
		(CC + GC)/GG	81.5/18.5	83.6/16.4	1.15 (0.75−1.76)	0.51
	**rs112561866**	GA	0.0	0.3	_	0.24
	**rs7974459**	CC/TC/TT	40.5/43.7/15.8	39.6/41.0/19.4	1.04 (0.72−1.50)	0.54
		CC/(TC + TT)	40.5/59.5	39.6/60.4	0.97 (0.69−1.35)	0.84
		(CC + TC)/TT	84.2/15.8	80.7/19.3	0.79 (0.51−1.21)	0.28
***KCNA2***	**rs3887820**	CC/CA/AA	79.8/18.5/1.7	82.9/15.4/1.7	_	0.6
		CC/(CA + AA)	79.8/20.2	82.9/17.1	1.23 (0.81−1.87)	0.33
		(CC + CA)/AA	98.3/1.7	98.3/1.7	1.00 (0.29−3.50)	1
***KCNV2***	**rs7029012**	GG/GC/CC	37.7/47.4/14.9	37/47.5/15.5	0.94 (0.66−1.35)	0.98
		GG/(GC + CC)	37.7/62.3	37/63	1.02(0.73−1.43)	0.91
		(GG + GC)/CC	85.1/14.9	84.5/15.5	1.04(0.66−1.63)	0.87
	**rs10967705**	GG/CG/CC	34.4/50.3/15.3	35.5/49.1/15.4	_	0.95
		GG/(CG + CC)	34.3/65.7	35.5/64.5	1.05 (0.75−1.47)	0.78
		(GG + CG)/CC	84.7/15.3	84.6/15.4	0.99 (0.64−1.55)	0.98
	**rs10967728**	GG/GC/CC	31.0/48.3/20.7	27.8/49.6/22.6	_	0.67
		GG/(GC + CC)	31.0/69.0	27.8/72.2	0.85 (0.60−1.22)	0.39
		(GG + GC)/CC	79.3/20.7	77.4/22.6	0.89 (0.60−1.33)	0.58

* Chi-Square Test with *p-*value < 0.05 is considered significant. OR: odds ratio; CI: confidence interval.

**Table 2:** The distributions of potassium channel related genes SNPs in the 172 generalized epileptic patients and 299 healthy controls.

Gene	SNP ID	Model	Cases (%)	Controls (%)	OR (95% CI)	*p*-Value *
***KCNA1***	**rs2227910**	CC/GC/GG	40.0/42.9/17.1	38.6/45.0/16.4	0.92 (0.61−1.39)	0.91
		CC/(GC + GG)	40.0/60.0	38.6/61.4	0.94 (0.64−1.39)	0.76
		(CC + GC)/GG	82.9/19.1	83.6/16.4	1.05 (0.63−1.73)	0.86
	**rs112561866**	GA	0.0 (0.0)	1.0 (0.3)	0.00 (0.00-NA)	0.34
	**rs7974459**	CC/TC/TT	38.3/43.7/18.0	39.6/41.1/19.3	1.10 (0.72−1.68)	0.85
		CC/(TC + TT)	38.3/61.7	39.6/60.4	1.06 (0.71−1.56)	0.78
		(CC + TC)/TT	82.0/18.0	80.7/19.3	0.92 (0.56−1.50)	0.73
***KCNA2***	**rs3887820**	CC/CA/AA	50.6/47.0/2.4	82.9/15.4/1.7	5.02 (3.23−7.80)	<0.0001
		CC/(CA + AA)	50.6/49.4	82.9/17.1	4.75 (3.09−7.28)	<0.0001
		(CC + CA)/AA	97.7/2.3	98.3/1.7	1.39 (0.37−5.24)	0.63
***KCNV2***	**rs7029012**	GG/GC/CC	39.3/47.6/13.1	34.8/49.3/15.9	0.86 (0.57−1.29)	0.54
		GG/(GC + CC)	39.3/60.7	34.8/65.2	0.82 (0.56−1.22)	0.34
		(GC + GG)/CC	86.9/13.1	84.1/15.9	0.80 (0.46−1.38)	0.41
	**rs10967705**	GG/CG/CC	34.5/50.9/14.6	35.5/49.2/15.3	1.06 (0.70−1.61)	0.94
		GG/(CG + CC)	34.5/65.5	35.5/64.5	1.04 (0.70−1.55)	0.84
		(GG + CG)/CC	85.4/14.6	84.6/15.4	0.94 (0.56−1.60)	0.82
	**rs10967728**	GG/GC/CC	30.8/49.7/19.5	27.8/49.6/22.6	0.90 (0.58−1.40)	0.67
		GG/(GC + CC)	30.8/69.2	27.8/72.2	0.87 (0.57−1.31)	0.5
		(GG + GC)/CC	80.5/19.5	77.4/22.6	0.83 (0.52−1.33)	0.44

* Chi-Square Test with *p*value < 0.05 is considered significant.

**Table 3:** Distribution of potassium channel related SNPs in the 172 generalized epileptic patients and 299 controls.

Epilepsy Subtype	SNP ID	Allele/Genotype	Cases *N* (%)	Controls *N* (%)	OR (95% CI)	*p*-Value *
**GM**	**rs7029012**	C	41 (36)	240 (41)	1.00	0.15
		G	73 (64)	352 (59)		
		GG	20 (35)	103 (35)	0.99 (0.54−1.79)	0.97
		CG + CC	37 (65)	193 (65)		
**GM**	**rs10967705**	C	44 (39)	239 (40)	1.00	0.51
		G	70 (61)	359 (60)		
		GG	19 (33)	106 (35)	1.10 (0.60−2.00)	0.76
		CG + CC	38 (67)	193 (65)		
**GM**	**rs3887820**	C	55 (48)	531 (91)	1.00	<0.0001
		A	59 (52)	55 (9)		
		AA	2 (4)	10 (1.7)	1.00	<0.0001
		CC + CA	55 (96)	288 (98.3)		
**GTC**	**rs10967728**	G	125 (56)	303 (53)	1.00	0.72
		C	99 (44)	273 (47)		
		GG	35 (31)	80 (28)	0.85 (0.53−1.36)	0.49
		GC + CC	77 (69)	208 (72)		
**GTC**	**rs10967705**	G	135 (59)	359 (60)	1.00	0.95
		C	93 (41)	239 (40)		
		GG	40 (35)	106 (35)	1.00	0.94
		GC + CC	74 (65)	193 (65)		

* Chi-Square Test with *p-*value < 0.05 is considered significant. GM: generalized myoclonic seizures, GTC: generalized tonic-clonic seizures.

**Table 4:** Distribution of potassium channel related SNPs in the 124 partial epileptic patients and 299 healthy controls.

Gene	SNP ID	Model	Cases (%)	Controls (%)	OR (95% CI)	*p*-Value *
***KCNA1***	**rs2227910**	CC/GC/GG	42.6/36.9/20.5	38.6/45.0/16.4	0.74 (0.46−1.19)	0.29
		CC/(GC + GG)	42.6/57.4	38.6/61.4	0.85 (0.55−1.30)	0.44
		(CC + GC)/GG	79.5/20.5	83.6/16.4	1.31 (0.77−2.24)	0.33
	**rs112561866**	GA	0.0 (0.0)	1.0 (0.3)	0.00 (0.00–NA)	0.4
	**rs7974459**	CC/TC/TT	43.6/43.6/12.8	39.6/41.1/19.3	0.97 (0.61−1.54)	0.28
		CC/(TC + TT)	43.6/56.4	39.6/60.4	0.85 (0.55−1.31)	0.47
		(CC + TC)/TT	87.2/12.8	80.7/19.3	0.61 (0.33−1.14)	0.11
***KCNV2***	**rs7029012**	GG/GC/CC	41.0/42.6/16.4	34.8/49.3/15.9	0.73 (0.46−1.17)	0.42
		GG/(GC + CC)	41.0/59.0	34.8/65.2	0.77 (0.50−1.18)	0.23
		(GC + GG)/CC	83.6/16.4	84.1/15.9	1.04 (0.59−1.84)	0.9
	**rs10967705**	GG/CG/CC	34.1/49.6/16.3	35.5/49.1/15.4	1.05 (0.66−1.67)	0.96
		GG/(CG + CC)	34.2/65.8	35.5/64.5	1.06 (0.68−1.65)	0.8
		(GG + CG)/CC	83.7/16.3	84.6/15.4	1.07 (0.60−1.89)	0.82
	**rs10967728**	GG/GC/CC	31.4/46.3/22.3	27.8/49.6/22.6	0.82 (0.50−1.35)	0.75
		GG/(GC + CC)	31.4/68.6	27.8/72.2	0.84 (0.53−1.33)	0.46
		(GG + GC)/CC	77.7/22.3	77.4/22.6	0.99 (0.59−1.64)	0.96

* Chi-Square Test with *p-*value < 0.05 is considered significant.

**Table 5:** Distribution of potassium channel related SNPs in the 134 poor responder and 162 good responder patients.

Gene	SNP ID	Model	Poor Responders (%)	Good Responders (%)	OR (95% CI)	*p*-Value
***KCNA1***	**rs2227910**	CC/GC/GG	40.2/46.2/13.6	41.9/35.6/22.5	1.35 (0.81−2.25)	0.07
		CC/(GC + GG)	40.1/59.9	41.9/58.1	0.54 (0.29−1.01)	0.77
		(CC + GC)/GG	86.4/13.6	77.5/22.5	1.07 (0.67−1.72)	0.05
	**rs7974459**	CC/TC/TT	40.6/45.3/14.1	40.4/42.3/17.3	1.06 (0.64−1.77)	0.73
		CC/(TC + TT)	40.6/59.4	40.4/59.6	0.99 (0.62−1.59)	0.97
		(CC + TC)/TT	85.9/14.1	82.7/17.3	0.78 (0.41−1.50)	0.45
***KCNA2***	**rs3887820**	CC/CA/AA	79.4/17.6/3.0	80.2/19.2/0.6	0.92 (0.51−1.67)	0.26
		CC/(CA + AA)	79.4/20.6	80.1/19.9	1.05 (0.59−1.86)	0.88
		(CC + CA)/AA	97.0/3.0	99.4/0.6	5.04 (0.56−45.60)	0.1
***KCNV2***	**rs7029012**	GG/GC/CC	40.5/43.5/16.0	39.6/47.2/13.2	0.90 (0.55−1.49)	0.73
		GG/(GC + CC)	40.5/59.5	39.6/60.4	0.97 (0.60−1.55)	0.89
		(GG + GC)/CC	84.0/16.0	86.8/13.2	1.25 (0.65−2.41)	0.5
	**rs10967705**	GG/CG/CC	37.3/45.5/17.2	31.9/54.4/13.7	0.72 (0.43−1.19)	0.31
		GG/(CG + CC)	37.3/62.7	31.9/68.1	0.79 (0.49−1.27)	0.33
		(GG + CG)/CC	82.8/17.2	86.2/13.8	1.30 (0.69−2.45)	0.42
	**rs10967728**	GG/GC/CC	30.5/45.8/23.7	31.4/50.3/18.3	0.94 (0.55−1.60)	0.51
		GG/(GC + CC)	30.5/69.5	31.5/68.5	1.04 (0.63−1.72)	0.87
		(GG + GC)/CC	76.3/23.7	81.8/18.2	1.39 (0.79−2.46)	0.26

* Chi-Square Test with *p-*value < 0.05 is considered significant.

## Supplementary Materials

The following are available online at http://www.mdpi.com/2075-4426/8/4/37/s1, Figure S1: A representative scatter plot of rs10967728 SNP from sequenom data, Table S1: SNP IDs, their position and genotyping data based on whole cohort (*N* = 595), Table S2: Demographic characteristics of 296 Jordanian unrelated epileptic patients, Table S3: Clinical characteristics of 296 Jordanian unrelated epileptic patients, Table S4: Demographic characteristics of 299 Jordanian unrelated healthy individuals, Table S5: Characteristics of selected SNPs of potassium channel related genes, Table S6: Frequencies of the haplotypes of *KCNA1* and *KCNV2* genes in the 172 generalized epileptic patients and 299 healthy controls, Table S7: Frequencies of the haplotypes of *KCNA1* and *KCNV2* genes in the 134 poor responder patients and 162 good responder patients.

## References

[1] World Health Organization (2001). Epilepsy: Aetiology, Epidemiology and Prognosis. http://www.who.int/news-room/fact-sheets/detail/epilepsy.

[2] Steinlein O.K. (2004). Genetic mechanisms that underlie epilepsy. Nat. Rev. Neurosci..

[3] Fisher R.S., van Emde Boas W., Blume W., Elger C., Genton P., Lee P., Engel J. (2005). Epileptic seizures and epilepsy: Definitions proposed by the International League against Epilepsy (ILAE) and the International Bureau for Epilepsy (IBE). Epilepsia.

[4] Nordli D.R. (2005). Idiopathic generalized epilepsies recognized by the International League against Epilepsy. Epilepsia.

[5] Prasad A.N., Prasad C., Stafstrom C.E. (1999). Recent advances in the genetics of epilepsy: Insights from human and animal studies. Epilepsia.

[6] D’Adamo M.C., Catacuzzeno L., Di Giovanni G., Franciolini F., Pessia M. (2013). K^+^ channelepsy: Progress in the neurobiology of potassium channels and epilepsy. Front. Cell. Neurosci..

[7] Camerino D.C., Tricarico D., Desaphy J.F. (2007). Ion channel pharmacology. Neurotherapeutics.

[8] Shah N.H., Aizenman E. (2014). Voltage-gated potassium channels at the crossroads of neuronal function, ischemic tolerance, and neurodegeneration. Transl. Stroke Res..

[9] Qu J., Lu S., Lu Z., Xu P., Xiang D., Qu Q. (2017). Pharmacogenetic and case–control study on potassium channel related gene variants and genetic generalized epilepsy. Medicine.

[10] Villa C., Combi R. (2016). Potassium channels and human epileptic phenotypes: An updated overview. Front. Cell. Neurosci..

[11] Kohling R., Wolfart J. (2016). Potassium channels in epilepsy. Cold Spring Harb. Perspect. Med..

[12] Zuberi S.M., Eunson L.H., Spauschus A., De Silva R., Tolmie J., Wood N.W., McWilliam R.C., Stephenson J.B., Kullmann D.M., Hanna M.G. (1999). A novel mutation in the human voltage-gated potassium channel gene (Kv1.1) associates with episodic ataxia type 1 and sometimes with partial epilepsy. Brain.

[13] Allen N.M., Conroy J., Shahwan A., Lynch B., Correa R.G., Pena S.D., McCreary D., Magalhães T.R., Ennis S., Lynch S.A. (2016). Unexplained early onset epileptic encephalopathy: Exome screening and phenotype expansion. Epilepsia.

[14] Syrbe S., Hedrich U.B., Riesch E., Djémié T., Müller S., Møller R.S., Maher B., Hernandez-Hernandez L., Synofzik M., Caglayan H.S. (2015). De novo loss- or gain-of-function mutations in *KCNA2* cause epileptic encephalopathy. Nat. Genet..

[15] Brew H.M., Gittelman J.X., Silverstein R.S., Hanks T.D., Demas V.P., Robinson L.C., Robbins C.A., McKee-Johnson J., Chiu S.Y., Messing A. (2007). Seizures and reduced life span in mice lacking the potassium channel subunit Kv1.2, but hypoexcitability and enlarged Kv1 currents in auditory neurons. J. Neurophysiol..

[16] Jorge B.S., Campbell C.M., Miller A.R., Rutter E.D., Gurnett C.A., Vanoye C.G., George A.L., Kearney J.A. (2011). Voltage-gated potassium channel KCNV2 (Kv8.2) contributes to epilepsy susceptibility. Proc. Natl. Acad. Sci. USA.

[17] Nakajima Y., Saito Y., Shiseki K., Fukushima-Uesaka H., Hasegawa R., Ozawa S., Sugai K., Katoh M., Saitoh O., Ohnuma T. (2005). Haplotype structures of EPHX1 and their effects on the metabolism of carbamazepine-10,11-epoxide in Japanese epileptic patients. Eur. J. Clin. Pharmacol..

[18] Johnson M.R., Tan N.C., Kwan P., Brodie M.J. (2011). Newly diagnosed epilepsy and pharmacogenomics research: A step in the right direction. Epilepsy Behav..

[19] Kasperaviciute D., Sisodiya SM. (2009). Epilepsy pharmacogenetics. Pharmacogenomics.

[20] Qu J., Zhou B.T., Yin J.Y., Xu X.J., Zhao Y.C., Lei G.H., Tang Q., Zhou H.H., Liu Z.Q. (2012). *ABCC2* polymorphisms and haplotype are associated with drug resistance in Chinese epileptic patients. CNS Neurosci. Ther..

[21] Zhou B.T., Zhou Q.H., Yin J.Y., Li G.L., Qu J., Xu X.J., Liu D., Zhou H.H., Liu Z.Q. (2012). Effects of SCN1A and GABA receptor genetic polymorphisms on carbamazepine tolerability and efficacy in Chinese patients with partial seizures: 2-year longitudinal clinical follow-up. CNS Neurosci. Ther..

[22] Ma C.L., Wu X.Y., Zheng J., Wu Z.Y., Hong Z., Zhong M.K. (2014). Association of *SCN1A*, *SCN2A* and *ABCC2* gene polymorphisms with the response to antiepileptic drugs in Chinese Han patients with epilepsy. Pharmacogenomics.

[23] Steffens M., Leu C., Ruppert A.K., Zara F., Striano P., Robbiano A., Capovilla G., Tinuper P., EPICURE Consortium, EMINet Consortium (2012). Genome-wide association analysis of genetic generalized epilepsies implicates susceptibility loci at 1q43, 2p16.1, 2q22.3 and 17q21.32. Hum. Mol. Genet..

[24] Kimchi-Sarfaty C., Oh J.M., Kim I.W., Sauna Z.E., Calcagno A.M., Ambudkar S.V., Gottesman M.M. (2007). A “silent” polymorphism in the *MDR1* gene changes substrate specificity. Science.

[25] Fung K.L., Pan J., Ohnuma S., Lund P.E., Pixley J.N., Kimchi-Sarfaty C., Ambudkar S.V., Gottesman M.M. (2014). MDR1 synonymous polymorphisms alter transporter specificity and protein stability in a stable epithelial monolayer. Cancer Res..

[26] Eunson L., Rea R., Zuberi S., Youroukos S., Panayiotopoulos C., Liguori R., Avoni P., McWilliam R., Stephenson J., Hanna M. (2000). Clinical, genetic, and expression studies of mutations in the potassium channel gene *KCNA1* reveal new phenotypic variability. Ann. Neurol..

