# Supplementary material for: The Impact of Potassium Channel Gene Polymorphisms on Antiepileptic Drug Responsiveness in Arab Patients with Epilepsy
Source: J Pers Med. 2018 Nov 14;8(4):37. doi: 10.3390/jpm8040037 (PMC6313615; doi:10.3390/jpm8040037)
Supplement: Supplementary file 1 [file jpm-08-00037-s001.pdf]

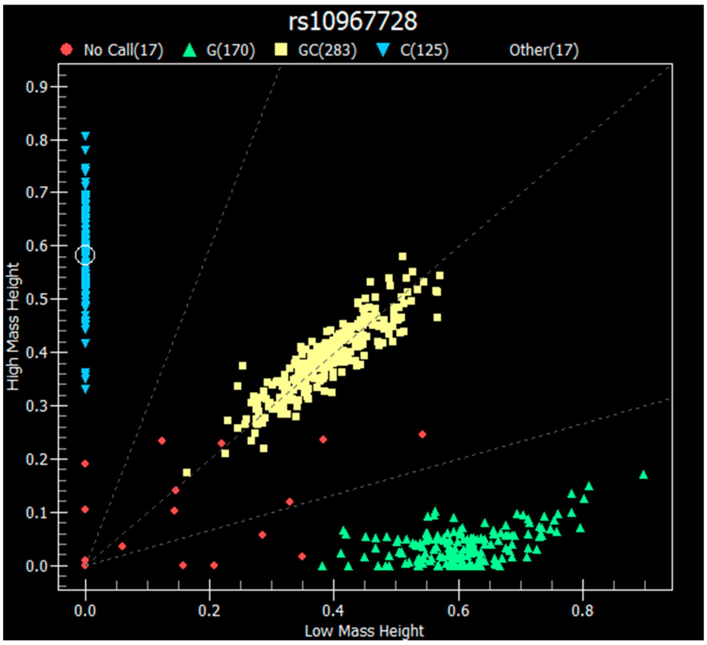

Figure S1: A representative scatter plot of rs10967728 SNP from sequenom data. The scatter plot illustrates rs10967728 (G>C) SNP within *KCNV2* gene. The X and Y axes denote mass measurements for the two alleles (G, C, low mass allele versus high mass allele) at the rs10967728 SNP. Every single sample is represented by one point. The points in the scatter plot are colored depending on the genotype calls

Table S1: SNP IDs, their position and genotyping data based on whole cohort (N = 595)						
**Genes**	**SNP ID**	**SNP**	**Chr Positions**	**SNP type**	**Discrepancy rate[b]**	**Call rate[c]**
KCNA1	**rs2227910**	C/G	12:4912182	Synonymous codon	0.02%	98%
	**rs112561866**	G/A	12:4912725	Missense (p.Met449Ile)	0.01%	99%
	**rs7974459**	T/C	12:4914547	3' UTR variant	0.06%	95%
KCNA2	**rs3887820**	G/T	1:110605458	5' UTR variant	0.02%	98%
KCNV2	**rs7029012**	C/G	9:2717698	Transcript variant, 5' UTR variant	0.03%	98%
	**rs10967705**	C/G	9:27117922	Transcript variant, synonymous codon	0.01%	99%
	**rs10967728**	G/C	9:2721794	Intronic variant	0.05%	96%

a. Chromosome positions are based on NCBI Human Genome Assembly Build.
b. Ratio of the number of discordant genotypes to the number of duplicates.
c. Ratio of the number of valid genotypes to the number of subjects genotyped (N = 595) at each locus

**Table S2:** Demographic characteristics of 296 Jordanian unrelated epileptic patients			
**Category**	**Subcategory**	**Good responder**	**Poor responder**
**Demographics**	Patients (N, %)	(162/296) 54.7%	(134/296) 45.3%
	Age[a] [years]	7.5 [4.0]	6.6 [4.1]
	BMI[a]	18.5 [10.0]	17.0 [6.6]
	Male	56.2%	53.0%
	Female	43.8%	47.0%
**Age at onset**	Age[a] [years]	3.5 [3.4]	3.3 [3.2]
**Classification of Epileptic seizure**	EGS[b]	53. 7%	65.7%
	EPS[c]	46.3%	34.3%
**Subgroups of EGS**	GM[d]	19.8%	46.5%
	GTC[e]	80.2%	53.5%

[a] Mean Standard deviation in square brackets
[b] EGS: Epileptic Generalized Seizure
[c] EPS:Epileptic Partial Seizure
[d] GM: Generalized Myoclonic
[e] GTC: Generalized Tonic Colonic

**Table S3:** Clinical characteristics of 296 Jordanian unrelated epileptic patients			
**Category**	**Subcategory**	**Good responder**	**Poor responder**
**Clinical**	Patients (N, %)	(162/296) 54.7%	(134/296) 45.3%
	Family history (N, %)	(49/162) 30.2%	(36/134) 26.9%
	Non- epilepsy related co-morbidity (N, %)	(7/162) 4.3%	(9/134) 6.7%
	Psychosis (N, %)	(5/162) 3.1%	(8/134) 6.0%
	Suicidal thought or action (N, %)	(1/162) 0.6%	(1/1134) 0.7%

**Table S4:** Demographic characteristics of 299 Jordanian unrelated healthy individuals		
**Category**	**Subcategory**	**Control**
**Demographic**	Age[a] [years]	5.9 [3.8]
	BMI[a]	16.7 [11.2]
	Male	50.8%
	Female	49.2%

[a] Mean Standard deviation in square brackets

**Table S5:** Characteristics of selected SNPs of potassium channel related genes			
**Genes**	**rs numbers**	**Global MAF**	**Current Study MAF**
KCNA1	rs2227910	G=0.4667/2337	G=0.39
	rs112561866	A=0.00002/3	A=0.0
	rs7974459	T=0.4824/2416	T=0.39
KCNA2	rs3887820	A=0.2845/1425	A=0.1
KCNV2	rs7029012	C=0.3281/1643	NA*
	rs10967705	C=0.3397/1701	C=0.4
	rs10967728	C=0.4860/2434	C=0.46

*NA: not applicable
MAF: major allele frequency

**Table S6:** Frequencies of the haplotypes of *KCNA1* and *KCNV2* genes in the 172 generalized epileptic patients and 299 healthy controls					
**Gene**	**Haplotypes**	**GEs (%)**	**Controls (%)**	**Odds ratio (95% CI)**	**P value**
KCNA1	CGT	0.034	0.048	0.71 (0.39 - 1.30)	0.27
	CGC	0.582	0.562	1.00	_
	GGT	0.346	0.347	0.97 (0.77 - 1.23)	0.8
KCNV2	CCG	0.319	0.313	0.99 (0.72 - 1.37)	0.96
	GGG	0.201	0.188	(0.69 - 1.52)	0.91
	GGC	0.389	0.383	1.00	_

*p value < 0.05 is considered significant

**Table S7:** Frequencies of the haplotypes of *KCNA1* and *KCNV2* genes in the 134 poor responder patients and 162 good responder patients					
**Gene**	**Haplotypes**	**Poor responder (%)**	**Good responder (%)**	**Odds ratio (95% CI)**	**P value**
KCNA1	CT	0.039	0.029	1.23 (0.50 - 3.05)	0.65
	CC	0.598	0.569	1.00	_
	GT	0.337	0.355	0.92 (0.66 - 1.29)	0.64
KCNV2	CCG	0.328	0.327	0.96 (0.65 - 1.42)	0.85
	GGG	0.148	0.199	0.92 (0.58 - 1.44)	0.71
	GGC	0.394	0.389	1.00	_

*p value < 0.05 is considered significant
